# Causes, costs and consequences of kinesin motors communicating through the microtubule lattice

**DOI:** 10.1242/jcs.260735

**Published:** 2023-03-03

**Authors:** Kristen J. Verhey, Ryoma Ohi

**Affiliations:** Department of Cell & Developmental Biology, University of Michigan Medical School, Ann Arbor, MI 48109, USA

**Keywords:** Kinesin, Microtubule, Tubulin, Microtubule lattice, Tubulin code, GTP island, Microtubule repair

## Abstract

Microtubules are critical for a variety of important functions in eukaryotic cells. During intracellular trafficking, molecular motor proteins of the kinesin superfamily drive the transport of cellular cargoes by stepping processively along the microtubule surface. Traditionally, the microtubule has been viewed as simply a track for kinesin motility. New work is challenging this classic view by showing that kinesin-1 and kinesin-4 proteins can induce conformational changes in tubulin subunits while they are stepping. These conformational changes appear to propagate along the microtubule such that the kinesins can work allosterically through the lattice to influence other proteins on the same track. Thus, the microtubule is a plastic medium through which motors and other microtubule-associated proteins (MAPs) can communicate. Furthermore, stepping kinesin-1 can damage the microtubule lattice. Damage can be repaired by the incorporation of new tubulin subunits, but too much damage leads to microtubule breakage and disassembly. Thus, the addition and loss of tubulin subunits are not restricted to the ends of the microtubule filament but rather, the lattice itself undergoes continuous repair and remodeling. This work leads to a new understanding of how kinesin motors and their microtubule tracks engage in allosteric interactions that are critical for normal cell physiology.

## Teaser

**Summary:** Kinesins can alter the microtubule track while they are walking on it. Thus, the microtubule is not simply a track for kinesin motility but a medium through which motors and microtubules can influence each other.

## Introduction

**Fig. 1.**
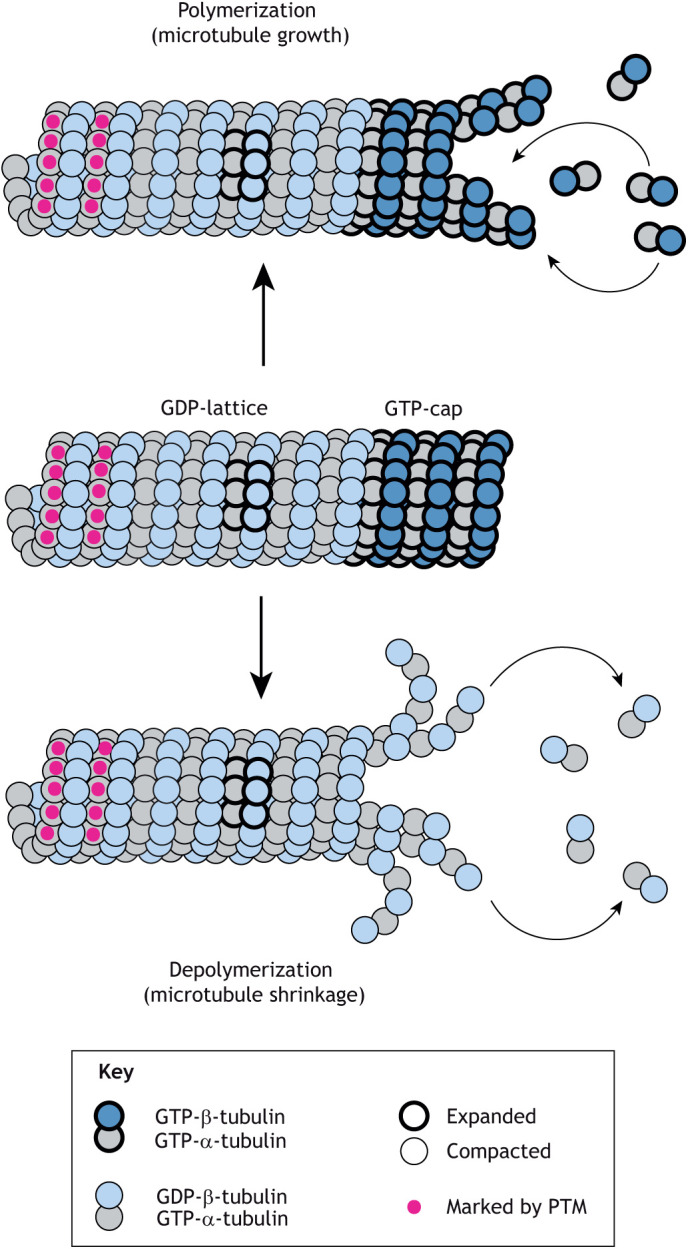
**Microtubule dynamics.** Tubulin subunits (dimers of α- and β-tubulin) self-assemble in a head-to-tail fashion to form a cylindrical microtubule. Tubulin subunits with GTP bound to β-tubulin (GTP-tubulin, dark blue) can undergo polymerization (microtubule growth), whereas tubulin subunits with GDP bound to β-tubulin (GDP-tubulin, light blue) fall off the polymer (microtubule shrinkage). GTP hydrolysis drives a structural change in tubulin from an expanded conformation (thick outline) to a compacted conformation (thin outline). Tubulin subunits within the GDP-lattice can be altered by posttranslational modifications (PTMs, magenta circles) or by adopting the expanded conformation (thick outline).

Microtubules are a major cytoskeletal polymer that is critical for a variety of important functions in eukaryotic cells, from structural support to cell division to movement within and of cells. The building block of the microtubule is the protein tubulin, a heterodimer of α- and β-tubulin. Tubulin subunits self-assemble in a head-to-tail fashion to make linear and polar protofilaments. Thirteen protofilaments bind laterally and close to form the cylindrical microtubule. The classic view of microtubule dynamics is that tubulin subunits add to and dissociate from one end, the ‘plus’ or growing end, and switch stochastically between periods of growth (polymerization) and shrinkage (depolymerization) (Fig. 1; reviewed in Cleary and Hancock, 2021; Gudimchuk and McIntosh, 2021). This property, termed dynamic instability, is driven by hydrolysis of GTP that is bound to the β-tubulin subunit. Soluble tubulin subunits are GTP bound and can undergo polymerization. GTP hydrolysis occurs shortly after a tubulin subunit incorporates into the microtubule. Microtubules composed of GTP-tubulin are stable, as best illustrated by the long-lived nature of microtubules assembled with slowly hydrolyzable GTP analogs [e.g. GMPCPP (Hyman et al., 1992)]. In contrast, microtubules composed of GDP-tubulin are unstable and disassemble rapidly. Thus, the process of microtubule assembly creates a filament with an unstable GDP-lattice and a stable ‘GTP-cap’. If polymerization slows or stops, the GTP-cap is lost, and the microtubule depolymerizes (Fig. 1).

Recent cryo-electron microscopy (cryo-EM) and X-ray fiber diffraction studies have provided clues as to how hydrolysis of the bound GTP by β-tubulin drives dynamic instability. For vertebrate microtubules, GTP hydrolysis and/or phosphate release result in conformational changes that twist and shorten (by ∼2 Å/subunit) the microtubule lattice and thus alter tubulin–tubulin interactions between protofilaments (lateral interactions) and along the protofilament axis (longitudinal interactions) (Alushin et al., 2014; Kamimura et al., 2016; LaFrance et al., 2022; Manka and Moores, 2018; Zhang et al., 2015, 2018). Thus, a lattice containing GTP-tubulin (i.e. GMPCPP-tubulin) subunits is in an ‘expanded’ state (∼8.4 nm/tubulin) whereas a lattice containing GDP-tubulin subunits is in a ‘compacted’ state (∼8.2 nm/tubulin) (Fig. 1). One caveat is that some studies examined microtubules decorated with monomeric kinesin-1 motor domain (e.g. Alushin et al., 2014), which is now known to change lattice structure (discussed below). Other studies used the microtubule-stabilizing agent taxol; however, the expanded state of GDP-taxol microtubules does not appear to mimic that of GMPCPP-tubulin-containing microtubules (hereafter GMPCPP-microtubules) (Yajima et al., 2012; Alushin et al., 2014; Kellogg et al., 2017; Manka and Moores, 2018), suggesting that the full range of tubulin conformations in the lattice is not yet known.

Although all microtubules are polymerized from tubulin subunits, cells can generate heterogenous microtubule populations that differ biochemically. For example, segments of GTP-tubulin (i.e. GTP islands) can be observed within the lattice of GDP-microtubules (Fig. 1, thick outline) using the hMB11 antibody (Dimitrov et al., 2008). In addition, tubulin subunits within the lattice can be marked by post-translational modifications (PTMs) (Fig. 1, magenta circles) that are posited to encrypt spatial, temporal and functional information important for specific microtubule functions. This concept, termed the ‘tubulin code’, is analogous to the ‘histone code’, which posits that histone PTMs regulate the functional properties of chromatin (Verhey and Gaertig, 2007). Much current work is focused on how the tubulin code is spatially and temporally deposited by ‘writer’ enzymes, translated into microtubule outputs by ‘reader’ proteins and removed by ‘eraser’ enzymes (Janke and Magiera, 2020; Roll-Mecak, 2020).

**Fig. 2.**
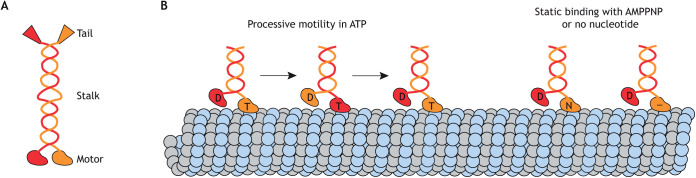
**Kinesin structure and motility.** (A) General schematic of a dimeric kinesin motor protein showing the motor, stalk and tail domains. (B) Processive motility of a truncated (constitutively active) kinesin along the microtubule surface. Alternating ATP hydrolysis by the two motor domains enables stepping (movement from one tubulin subunit to the next) along a protofilament of a microtubule. In the absence of nucleotide or in the presence of the non-hydrolyzable analog AMPPNP, kinesins bind statically to the microtubule surface. T, ATP; D, ADP; N, AMPPNP; −, no nucleotide.

A large variety of motile and non-motile microtubule-associated proteins (MAPs) serve to spatially and temporally regulate the nucleation, polymerization, organization and output of microtubules. Kinesins are a superfamily of MAPs that all have a kinesin motor domain (∼350 amino acids, Fig. 2A) that binds both ATP and tubulin. For motile kinesins, ATP hydrolysis triggers conformational changes in the kinesin motor domain that result in directed motion of the kinesin protein along a microtubule track. Kinesin proteins are placed within families based on sequence conservation within the kinesin motor domain (Miki et al., 2001; Wickstead and Gull, 2006; Wickstead et al., 2010). Outside of the motor domain, each kinesin protein contains unique stalk and tail sequences for oligomerization, binding specific cellular cargoes, and regulating motor activity (Fig. 2A) (Hirokawa et al., 2009; Verhey and Hammond, 2009).

The first kinesin to be discovered was kinesin-1 [originally referred to as conventional kinesin (Brady, 1985; Vale et al., 1985); encoded by *KIF5A*, *KIF5B* and *KIF5C* in humans] and consequently, it is the most-studied kinesin motor protein. Kinesin-1 is the archetypal transport kinesin and has been implicated in the transport and/or positioning of nearly every organelle in the cell. Enzymatic activity of the core motor domain enables kinesin-1 to move processively (i.e. to maintain an interaction with the microtubule for many rounds of catalysis) toward the plus-end of the microtubule in 8-nm steps (the distance between adjacent β-tubulin subunits) at speeds of 0.6–0.8 μm/s. Processivity is caused by a stepping mechanism in which the two motor domains of a dimeric kinesin alternate their catalytic cycles in order to maintain the motor–microtubule interaction (Fig. 2B; reviewed in Wang et al., 2015; Hancock, 2016; Hwang et al., 2017; Hwang and Karplus, 2019).

Processive motility that can drive cargo transport is a common feature of many kinesins, including those in the kinesin-2, kinesin-3 and kinesin-4 families (Verhey et al., 2011; Hunter and Allingham, 2020). Studies of processively stepping kinesin proteins have generally considered the microtubule to be simply a track that does not change over time. However, recent studies challenge this classic view by showing that motors and microtubules can influence each other, and the goal of this review is to consider this body of work. We first discuss kinesin-1 and its ability to not only read and respond to microtubule heterogeneity but also to induce heterogeneity via transient conformational changes within the microtubule lattice. The ability of kinesin-1 to induce conformational change is a double-edged sword as it enables through-the-lattice communication but at the expense of microtubule integrity. We then turn to recent work showing that kinesin-4 proteins also engage in through-the-lattice communication that impacts microtubule properties, as well as other motors and MAPs, over micrometer distances. We end by highlighting other examples of through-the-lattice allostery (regulation of distant sites) and discussing open questions for future research.

## Kinesin-1 as a reader and writer of the tubulin state within the microtubule lattice

### Kinesin-1 can ‘read’ the state of tubulin in the microtubule lattice

**Fig. 3.**
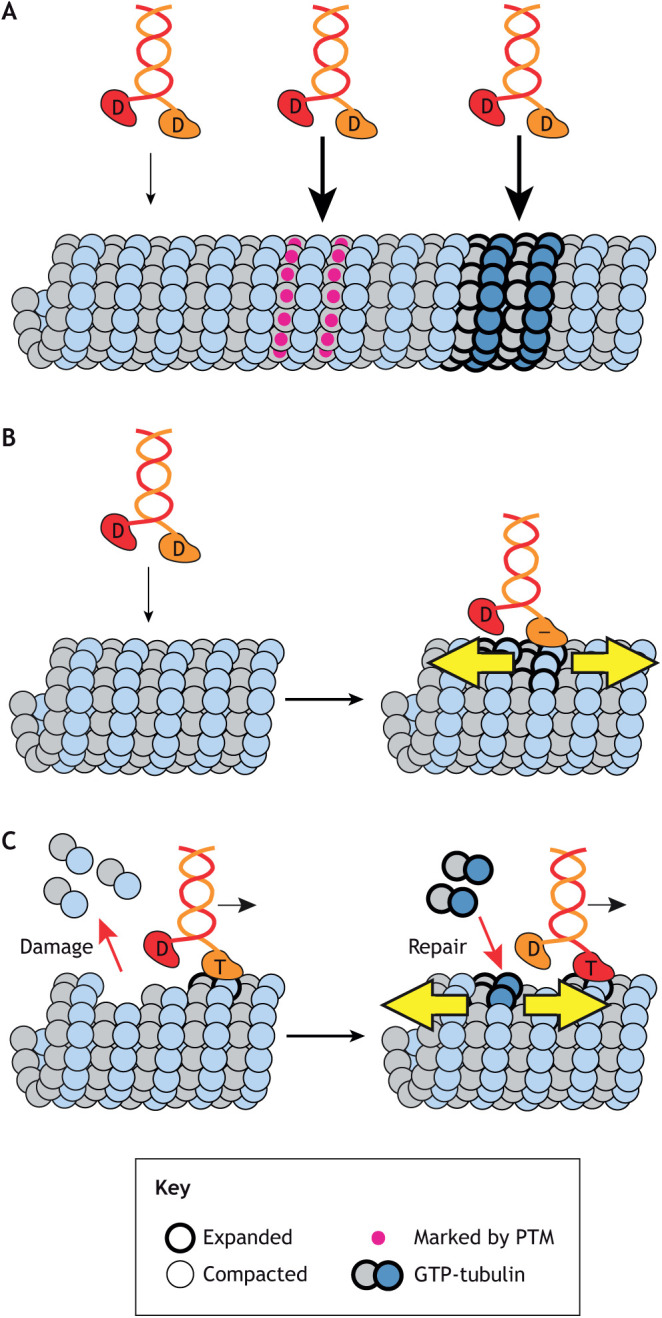
**Kinesin-1 can write and read the tubulin state in the microtubule lattice.** (A) Kinesin-1 reads the tubulin state and binds preferentially to microtubules containing tubulins marked by specific PTMs (magenta circles) or tubulins in the GTP-like/expanded state (thick outline). (B) Static kinesin-1 binding writes an expanded state to a GDP-lattice, enabling through-the-lattice allostery (yellow arrows) with other motors. (C) Walking kinesin-1 writes a GTP-tubulin (expanded) state by creating damage sites in the lattice that can be repaired by soluble GTP-tubulin.

During neuronal differentiation in primary hippocampal neurons, a constitutively active version of the kinesin-1 KIF5C was found to localize selectively to one neurite, and this neurite developed into the single axon of the cell (Nakata and Hirokawa, 2003; Jacobson et al., 2006; Reed et al., 2006). This selective transport results in localization of the kinesin-1 cargo protein JNK-interacting protein 1 (JIP1; also known as MAPK8IP1) to the developing axon (Reed et al., 2006). The mechanism of polarized trafficking was suggested to be due to preferential recognition of microtubules containing specific PTMs by kinesin-1. Specifically, in cells, the kinesin-1 motor domain was found to preferentially walk along microtubules marked by acetylation of α-tubulin at K40 (αTub-K40ac) and/or detyrosination of the C-terminal tail of α-tubulin (αTub-ΔY) (Liao and Gundersen, 1998; Reed et al., 2006; Dunn et al., 2008; Cai et al., 2009; Konishi and Setou, 2009). Thus, kinesin-1 can read the PTM state in that it preferentially binds to and steps along specific PTM-marked microtubule tracks (Fig. 3A).

Later work suggested that the ability of kinesin-1 to undergo preferential transport to the axon is due to its ability to respond to the nucleotide state of tubulin within the microtubule lattice. In developing axons, a rigor (immotile) mutant of KIF5C colocalized with the hMB11 antibody, which is thought to mark GTP islands (Nakata et al., 2011). In *in vitro* assays with purified proteins, the KIF5C motor domain displayed a higher affinity for GMPCPP-microtubules over GDP-tubulin-containing microtubules (hereafter GDP-microtubules) (Fig. 3A) (Nakata et al., 2011; Shima et al., 2018). However, this preferential binding was not observed for full-length kinesin-1 (Li et al., 2017), and it is yet unclear whether the differences between the studies are due to the use of truncated versus full-length kinesin, the source of tubulin, and/or the mechanism of GDP-microtubule stabilization for microscopy studies (i.e. glycerol versus taxol). It is also unclear whether the higher affinity for GMPCPP-microtubules reflects the ability of kinesin 1 to read the nucleotide state or the expanded state of the GMPCPP-microtubule lattice. Evidence for the latter comes from work on the immotile MAP tau, which forms cooperative assemblies on compacted GDP-lattice microtubules that repel kinesin-1 binding and motility (Siahaan et al., 2019, 2022). Thus, kinesin-1 can also read the GTP-like and/or expanded state of the microtubule lattice (Fig. 3A).

The recent work on microtubule lattice expansion and compaction raises the possibility that a microtubule lattice containing αTub-K40ac or αTub-ΔY tubulin is in an expanded state. If true, this finding would reconcile the seemingly disparate results between preferential binding of kinesin-1 to PTM-marked microtubules and to GTP-like microtubules. This finding would also reconcile the differences in the ability of kinesin-1 to recognize PTM-marked microtubules in cells (Reed et al., 2006; Dunn et al., 2008; Cai et al., 2009; Konishi and Setou, 2009; Guardia et al., 2016; Balabanian et al., 2017; Tas et al., 2017; Jansen et al., 2021 preprint) and its inability to distinguish marked and unmarked microtubules in *in vitro* assays (Kaul et al., 2014; Walter et al., 2012). Because the *in vitro* assays necessarily used taxol-stabilized microtubules, the effects of taxol on the microtubule lattice could obscure the ability of kinesin-1 to select marked over unmarked microtubules. Indeed, most *in vitro* studies of molecular motors have used microtubules stabilized by taxol to prevent microtubule depolymerization. In the future, it will be important for investigators to use microtubules that more closely mimic the physiological conditions of a GDP-lattice, for example, using a GMPCPP-cap to stabilize GDP-microtubules.

### Kinesin-1 binding can alter the conformation of tubulin within the microtubule lattice

Several groups have reported cooperative binding of kinesin-1 proteins in *in vitro* assays as some microtubules were found to have high level of kinesin-1 decoration and other areas had little or no decoration (Vilfan et al., 2001; Roos et al., 2008). Cooperative binding was also observed for beads carried by multiple kinesin-1 motors (Muto et al., 2005). These studies suggest that the microtubule has both low-affinity and high-affinity states for kinesin-1, and that binding of kinesin-1 causes structural changes in the microtubule lattice that propagate the high-affinity state over an extended length (Fig. 3B). Notably, these studies suggest that kinesin-1 can induce allosteric changes even in taxol-stabilized microtubules, consistent with early structural work (Krebs et al., 2004).

Recent work using a variety of single-molecule fluorescence microscopy and biophysical assays has shed light on the molecular basis for this intriguing finding. CryoEM reconstructions of motor-decorated microtubules showed that static binding of kinesin-1 (using the non-hydrolyzable ATP analog AMPPNP) allosterically triggers conformational changes in a GDP-tubulin subunit to a conformation that resembles GTP-tubulin (Shima et al., 2018). Furthermore, another study showed that static binding of kinesin-1 to GDP-microtubules increases their length by uniformly expanding the microtubule lattice along its axis (Peet et al., 2018). Measurements of lattice expansion indicate an elongation of ∼1.64Å per tubulin (Peet et al., 2018), which is less than the difference between compacted GDP-tubulin and expanded GMPCPP-tubulin (Alushin et al., 2014; Manka and Moores, 2018; Zhang et al., 2018). Thus, kinesin-1 binding appears to promote a semi-expanded state rather than one that mimics the fully expanded state of a GMPCPP lattice.

Molecular dynamics simulations indicate that the conformational changes in tubulin that are induced by kinesin-1 binding can occur very rapidly (on the order of 10 ns) and that GDP-tubulin will return elastically to its normal compacted conformation (Shi et al., 2021). Thus, the observed lattice expansion has necessarily been with kinesin-1 statically and strongly bound to a microtubule (i.e. in the absence of nucleotide or in the presence of AMPPNP; Fig. 2B) to capture the transient state. Collectively, these studies suggest that kinesin-1 binding can allosterically modify the conformation of GDP-tubulin (compacted with low affinity for kinesin-1) to give it properties that are more similar to those of GTP-tubulin (expanded with high affinity for kinesin-1). That is, kinesin-1 is thought to transiently ‘write’ a semi-expanded state into the lattice and thereby promote further kinesin-1 binding (Fig. 3B).

### Kinesin-1 stepping can stabilize or damage microtubules, depending on conditions

Observations of lattice expansion require high levels of microtubule decoration by static kinesin-1 motors, and it is not known whether a single kinesin-1 motor, or even small teams of kinesin-1, cause lattice expansion while walking along the microtubule during cargo transport. Surprisingly, in both single-molecule motility and gliding assays, walking kinesin-1 motors cause disassembly of GDP-microtubules (Triclin et al., 2021; Andreu-Carbo et al., 2022; Budaitis et al., 2022). In cells, kinesin-1-induced damage results in microtubules that break and fragment (Budaitis et al., 2022), indicating that motor-induced damage is physiologically relevant and might contribute to diseases that are driven by microtubule fragmentation, for example, neurological disorders (Dubey et al., 2015; Prokop, 2021).

How does kinesin-1 stepping cause damage to the lattice? It has been suggested that the physical act of kinesin-1 stepping from one tubulin subunit to the next causes the release of tubulin from the microtubule lattice, leaving a ‘hole’ or damage site (Fig. 3C) (Triclin et al., 2021). In support of this hypothesis, the addition of soluble tubulin in both gliding and single-molecule assays can prevent kinesin-1-induced microtubule disassembly by incorporating into the lattice and repairing the damage site (Triclin et al., 2021; Andreu-Carbo et al., 2022; Budaitis et al., 2022). Importantly, the amount of lattice damage and repair scale with the concentration of walking kinesin-1 molecules (Andreu-Carbo et al., 2022; Budaitis et al., 2022). It is important to reiterate that the use of GDP-microtubules allowed lattice damage to be observed. Indeed, taxol-GDP-microtubules are protected from motor-induced damage (Triclin et al., 2021; Budaitis et al., 2022), thus providing an explanation for why damage was not noted in previous gliding or single-molecule assays.

How does kinesin-1 stepping cause the release of tubulin from the microtubule lattice? One possibility is that the trailing motor domain ‘pulls out’ a tubulin subunit as it takes a forward step. Two lines of evidence currently argue against this possibility. First, single kinesin-1 motors generate forces significantly less than the ∼20 pN of force required to pull a tubulin subunit out of the lattice (Kuo et al., 2022). However, the ∼20 pN measurement was obtained under conditions where a pulling mechanism was applied to the α-tubulin C-terminus, which projects from the microtubule surface. While stepping, kinesin-1 interacts with the body of the tubulin subunit and alters its conformation (Shima et al., 2018). Thus, a walking kinesin-1 might require less force to pull out a tubulin subunit. Second, Andreu-Carbo et al. were unable to observe a tight temporal coupling between a walking kinesin-1 molecule and a repair event (incorporation of a new GTP-tubulin subunit; Andreu-Carbo et al., 2022). However, a lattice repair event is an indirect measurement of kinesin-1 damage as the lag time between lattice damage and the incorporation of a GTP-tubulin subunit from solution is not known.

An alternative possibility is that the ability of kinesin-1 to expand the lattice might simply accelerate the probability of a tubulin subunit spontaneously leaving the lattice. Although the spontaneous dissociation of lattice-embedded tubulin is very low, it does occur on a scale of minutes in *in vitro* assays (Schaedel et al., 2015; Reid et al., 2017; Schaedel et al., 2019). Kinesin-1-mediated expansion of one tubulin subunit causes steric clashes with neighboring tubulin subunits (Shima et al., 2018), which might increase the probability of tubulin dissociation from the microtubule lattice. These effects may be most acute at preexisting lattice defects, such as sites of missing tubulin subunits (vacancies) and/or shifts in the number of protofilaments. Such defects can be observed in microtubules assembled *in vitro,* as well as in cells, and represent energetically favorable sites for tubulin loss and re-addition (Chretien et al., 1992; Chretien and Fuller, 2000; Atherton et al., 2018, 2022; Chakraborty et al., 2020; Foster et al., 2022; Guyomar et al., 2022).

### What is the biological output of kinesin-1-induced lattice expansion and damage?

Walking kinesin-1 motors can generate a locally expanded microtubule lattice in two ways. First, binding of kinesin-1 causes a transient expansion of the GDP-tubulin subunits at the binding site (Fig. 3B). Second, walking kinesin-1 motors generate repair sites containing expanded GTP-tubulin subunits (Fig. 3C). If these structural changes are propagated along the lattice, this can result in a through-the-lattice coupling of motors and other MAPs. That is, walking kinesin-1 motors can cause the recruitment or exclusion of subsequent MAPs depending on their preference for an expanded versus compacted lattice, respectively. If the recruitment involves binding of subsequent kinesin-1 motors, this can create a positive feedback loop that designates a functional output for the microtubule. Indeed, recent work has suggested that walking kinesin-1 motors can generate preferred tracks that promote polarized transport (Shima et al., 2018; Andreu-Carbo et al., 2022).

The insertion of expanded GTP-tubulin subunits at sites of repair can also influence microtubule dynamics. These GTP-tubulin islands can serve as rescue sites during microtubule depolymerization (Dimitrov et al., 2008; Tropini et al., 2012; Aumeier et al., 2016; de Forges et al., 2016; Vemu et al., 2018; Schaedel et al., 2019; Bollinger et al., 2020; Rai et al., 2021), and thus a second output of kinesin-1-induced lattice damage could be an increase in microtubule length and overall density. Indeed, using dynamic microtubules, addition of kinesin-1 resulted in a concentration-dependent increase in rescue frequency and microtubule length (Andreu-Carbo et al., 2022; Budaitis et al., 2022).

However, the ability of kinesin-1 to expand and/or damage the lattice appears to be a double-edged sword. In the case of multiple motors attached to a bead, kinesins tend to pause or unbind from the microtubule when reaching a lattice defect (Liang et al., 2016). In addition, high levels of lattice damage in cells result in microtubules that are sensitive to mechanical stress and undergo breakage and fragmentation (Budaitis et al., 2022). These results indicate that kinesin-1-induced damage is physiologically relevant and might contribute to disease. These results also suggest that cells have likely evolved mechanisms to repair lattice damage and maintain their microtubule tracks.

In conclusion, evidence to date indicates that kinesin-1 proteins are capable of reading and responding to the PTM and/or expanded state of the microtubule lattice. Furthermore, kinesin-1 proteins can induce a positive feedback loop by writing conformational changes to tubulin subunits that can work allosterically through the lattice to influence microtubule dynamics and subsequent kinesin-1 binding. These positive effects are, however, shadowed by the ability of kinesin-1 to induce damage to the lattice that reduces microtubule integrity.

## Kinesin-4 motors also use through-the-lattice coupling to influence their motility

Members of the kinesin-4 family are known to walk processively to the plus-ends of microtubules and to influence microtubule dynamics. The *Xenopus* kinesin-4 Xklp1 slows down both microtubule polymerization and depolymerization in a concentration-dependent manner, resulting in static microtubules (Bringmann et al., 2004; Bieling et al., 2010). The effects of Xklp1 could be observed using monomeric and statically bound (in AMPPNP) motors, leading Bringmann and colleagues to postulate that the ability of Xklp1 to influence microtubule dynamics is due a change in microtubule structure induced by the presence of the bound motor. Consistent with this hypothesis, cryoEM of GDP-microtubules with statically bound Xklp1 showed that there were defects and break points along the lattice, frayed ends and regions of unwinding that could propagate along the microtubule (Bringmann et al., 2004). Thus, Xklp1-induced lattice changes appear to propagate to the microtubule ends and cause inhibition of dynamics.

**Fig. 4.**
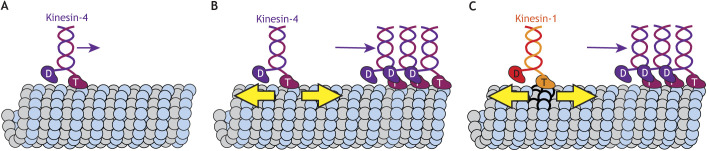
**Kinesin-1 and kinesin-4 motors communicate allosterically through the microtubule lattice.** (A) Individual kinesin-4 motors do not accumulate at microtubule ends because their run length is too short (small purple arrow). (B,C) While walking, (B) kinesin-4 and (C) kinesin-1 motors write changes to the microtubule that work allosterically through the lattice (yellow arrows) to influence other kinesin-4 motors. The increase in binding and motility of kinesin-4 (large purple arrow) enables its accumulation at microtubule ends.

That kinesin-4 motors cause structural changes that are propagated along the lattice is supported by recent work (Wijeratne et al., 2022). Like Xklp1, the homologous mammalian kinesin-4 protein KIF4A influences microtubule dynamics (Hu et al., 2011; Stumpff et al., 2012; Hannabuss et al., 2019; Mani et al., 2021). In addition, despite its ∼1 µm run length, KIF4A can accumulate at the plus ends of microtubules that are >10 µm long due to long-range, within-the-lattice coupling between KIF4A motors (Fig. 4A,B) (Wijeratne et al., 2022). Long-range coupling leads not only to increased motor binding and decreased unbinding but also to changes in the motility properties of KIF4A, including a concentration-dependent increase in processivity and decrease in speed that enable KIF4A accumulation at microtubule plus-ends (Wijeratne et al., 2022). The cooperative effects of KIF4A occur at low (pM) motor concentrations and affect other motors via long-range (several micrometers) interactions (Wijeratne et al., 2022). Thus, KIF4A motors can sense and respond to other motors bound far away on the microtubule lattice.

Taken together, these data suggest that kinesin-4 ‘writes’ structural changes into the microtubule lattice and this, in turn, can influence tubulin subunits at the microtubule ends as well as other motors on the microtubule surface. Like the coupling of kinesin-1 motors (Vilfan et al., 2001; Muto et al., 2005; Roos et al., 2008), the effects of KIF4A were observed on taxol-stabilized microtubules (Wijeratne et al., 2022), and it will be interesting to see how the effects play out on GDP-microtubules. The exact types of structural changes induced in the microtubule lattice by Xklp1 and KIF4A remain to be determined but might be similar to the tubulin expansion induced by walking kinesin-1 motors. Indeed, walking kinesin-1 motors ‘write’ a lattice state that is ‘read’ by KIF4A and results in an increase in processivity, reduction in unbinding rate and decrease in speed of KIF4A (Fig. 4C) (Wijeratne et al., 2022). These observations support the hypothesis that long-range interactions allow communication between motors that are separated on a length scale of micrometers.

## Other instances of long-range through-the-lattice coupling and their impacts

Are all molecular motors capable of inducing allosteric changes in tubulin subunits within the lattice or is this a property of only some motors? Although this review highlights the work on two plus-end-directed kinesins, there is emerging evidence that minus-end-directed motors can also impact the microtubule lattice while they are walking. First, stepping of the yeast proteins kinesin-14 Klp2 and cytoplasmic dynein causes microtubule breakage and disassembly that could be prevented by the inclusion of soluble tubulin to repair the damage site (Triclin et al., 2021). Second, a component of the axonemal dynein complex, the inner-arm dynein DNAH7, contains an 18-residue insertion that contacts an adjacent protofilament and induces large distortions in the microtubule cross-sectional curvature. This raises the possibility that dynein coordination in axonemes is mediated via conformational changes within the microtubule lattice (Lacey et al., 2019).

A number of non-motile MAPs have also been shown to alter the microtubule lattice upon binding. For example, end binding (EB; also known as MAPRE) proteins bind preferentially to GMPCPP- over GDP-microtubules, enabling them to track growing microtubule plus-ends. Once bound, EB proteins promote GTP hydrolysis and lattice compaction and can influence the activity of XMAP215-family microtubule polymerases via long-range, through-the-lattice effects (Zanic et al., 2009; Maurer et al., 2012; Zanic et al., 2013; Zhang et al., 2018; Reid et al., 2019; LaFrance et al., 2022). Thus, a MAP can ‘read’ one tubulin state and respond by ‘writing’ another tubulin state. A second example is the protein tau, which binds preferentially to GDP-microtubules and forms concentration-dependent cohesive ‘envelopes’ that stabilize the compacted GDP-lattice and negatively regulate kinesin-1 binding and motility, presumably via through-the-lattice effects (McVicker et al., 2011; Duan et al., 2017; Siahaan et al., 2019, 2022; Castle et al., 2020). Thus, a MAP can ‘read’ and stabilize the same tubulin state in the lattice.

Finally, long-range effects within the microtubule lattice can modulate the dynamics of the polymer itself, even in the absence of MAP binding and activity. In recent work, the concentration dependence of microtubule catastrophe observed in biochemical models could only be satisfied in computational modeling studies if long-range through-the-lattice interactions were included in the models (Kim and Rice, 2019).

Beyond microtubules, cooperative through-the-lattice coupling has been observed for various actin-binding proteins (ABPs) (reviewed in Tokuraku et al., 2020). For example, early work suggested that myosin-2 protein can ‘write’ structural changes in F-actin that can be cooperatively propagated through the actin filament (Orlova and Egelman, 1997). More recently, myosin-5 and myosin-6 proteins have been shown to ‘read’ different nucleotide states of actin subunits in the filament (Zimmermann et al., 2015). Recent advances that provide high-resolution cryo-EM structures of actin filaments in different nucleotide states (Oosterheert et al., 2022; Reynolds et al., 2022) provide a starting point for uncovering how motile and non-motile ABPs write and read lattice changes to drive cellular activities.

## Conclusions and open questions

Together, the results discussed above suggest that the kinesin-1 and kinesin-4 motors can ‘write’ transient structural changes to the microtubule lattice. We want to emphasize the transient nature of these motor-induced changes as compared to the more traditional view of ‘writing’ changes into the microtubule lattice that is carried out by enzymes that mark tubulin subunits with specific PTMs. Importantly, despite their transient nature, motor-driven conformational changes can impact microtubule properties, as well as other motors and MAPs, over micrometer distances. These findings have a number of implications for how we think about microtubules and their dynamics as well as molecular motors.

For microtubules, an important implication is the finding that addition and loss of tubulin subunits is not restricted to the ends of microtubules but also occurs along the lattice. The incorporation of new GTP-tubulin subunits along the lattice suggests a self-renewal process where ‘old’ subunits could be removed and replaced by ‘new’ subunits that impart new molecular and functional properties to the microtubule (Thery and Blanchoin, 2021). This process is likely to be particularly important in cells where microtubules span great lengths (i.e. neurons; Hahn et al., 2019; Pinho-Correia and Prokop, 2022) or are subjected to high levels of oxidative or other molecular stresses (e.g. cardiomyocytes; Goldblum et al., 2021; Uchida et al., 2022). A second important implication for microtubules is that different conformations of tubulin can override the nucleotide state in dictating biochemical interactions and rates in the lattice. As noted by Kim and Rice, through-the-lattice allostery allows the conformational state of one tubulin subunit to indirectly affect the biochemistry of distant (beyond nearest-neighbor) tubulins (Kim and Rice, 2019).

For molecular motors, an important implication is that the microtubule can no longer be considered a passive track on which the motors move, but rather, the microtubule is a plastic surface through which motors can communicate with each other and with other MAPs (Cross, 2019). A second implication for molecular motors is that most, if not all, motors are likely to impact microtubule dynamics. Previously, only members of the kinesin-8 and kinesin-13 families were thought to impact microtubule dynamics through their action at microtubule ends (Friel and Welburn, 2018; Lin et al., 2020). Recent work on kinesin-1 suggests that motors that can impact tubulin structure within the lattice might also facilitate tubulin loss from the lattice. Although the damage site can be repaired, too much damage is catastrophic to microtubule integrity (Triclin et al., 2021; Budaitis et al., 2022).

Studies of through-the-lattice coupling of motors and MAPs are just emerging and there are many interesting questions for the years ahead and below we highlight a few of those.

First, which motors and MAPs affect tubulin conformation within the lattice and at what concentrations, distances, and time scales? Through-the-lattice allostery seems to be a general feature of mammalian kinesin-1 proteins, and it also might be a general feature of the kinesin-4 family as other members (KIF7, KIF27, KIF21A, KIF21B and KLP-12) suppress microtubule dynamics (van der Vaart et al., 2013; He et al., 2014; Ghiretti et al., 2016; Muhia et al., 2016; van Riel et al., 2017; Yue et al., 2018; Taguchi et al., 2022). However, there are differences between kinesin-1 and the kinesin-4 motors as through-the-lattice effects can be observed at low motor concentrations for KIF4A (pM) versus those induced by kinesin-1 (nM). In addition, KIF4A-induced lattice changes impact events on a longer length scale than those of kinesin-1 (Wijeratne et al., 2022), suggesting that through-the-lattice communication from KIF4A involves a conformational switch in a large number of intervening tubulin subunits. Finally, the time scales at which kinesins alter tubulin conformation to impact downstream events are largely unexplored. Molecular modeling suggests that GDP-tubulin will return elastically to its normal compacted conformation on a time scale of seconds (Shi et al., 2021), yet the effect of kinesin-1 on microtubule elongation and subsequent motor binding lasted a few minutes (Shima et al., 2018). The persistence of the tubulin expanded state after kinesin-1 detaches raises the possibility of conformational memory in the microtubule lattice.

Second, which motors and MAPs respond to changes in the microtubule lattice and how do they respond? The most likely response of a motor or MAP is an increase in the initial binding (i.e. landing rate) to a microtubule lattice in a specific conformational state, although effects on unbinding or enzymatic properties have also been noted. For example, both kinesin-1 and KIF4A respond to the lattice state by increased binding and decreased unbinding, whereas KIF4A also shows changes in the run length, lifetime, and velocity (Wijeratne et al., 2022).

Third, what is the range of conformations adopted by tubulin subunits in the microtubule lattice? Recent high-resolution cryo-EM structures have provided insight into the average tubulin conformation in specific nucleotide, microtubule-targeting compound or MAP-bound states (Yajima et al., 2012; Alushin et al., 2014; Zhang et al., 2015, 2018; Kamimura et al., 2016; Kellogg et al., 2017; Manka and Moores, 2018; LaFrance et al., 2022; Estevez-Gallego et al., 2023), but we do not know the full range of conformations that tubulins can adopt within the lattice or which states are promoted by different motors and MAPs.

Fourth, how is lattice damage repaired? Although some motor-induced lattice damage can be beneficial through the generation of GTP islands, too much damage makes microtubules susceptible to mechanical stress (Budaitis et al., 2022). Thus, cells need to balance allowing critical motor-driven transport events and repairing the damage inflicted by these events. In experiments with purified components, free tubulin in solution is sufficient for lattice repair and can protect microtubules from breakage (Schaedel et al., 2015, 2019; Aumeier et al., 2016; de Forges et al., 2016; Triclin et al., 2021; Andreu-Carbo et al., 2022; Budaitis et al., 2022). However, in cells, lattice repair might be accelerated and regulated by specific proteins or complexes. If so, how is lattice damage recognized and is there a size limit for a damage site that can be repaired?

Finally, how do microtubule-targeting compounds protect the lattice from damage and breakage? Why taxol-treated microtubules are not broken down by walking kinesin-1 motors remains unclear (Triclin et al., 2021; Andreu-Carbo et al., 2022; Budaitis et al., 2022). It might be due to taxol reinforcing the links between tubulin dimers in the lattice, and thus preventing the occurrence of damage, or to the stabilization of links around sites of lattice damage. A number of clinically relevant compounds have been identified that also stabilize microtubules but their impacts on microtubule structure and lattice repair are largely unknown.

In closing, emerging work challenges the notion that microtubules are simply passive tracks for MAPs and molecular motor proteins. Here, we have summarized work showing that the structural state and integrity of the microtubule can be altered by MAPs and motors, and this in turn affects microtubule dynamics, intracellular transport, and the overall architecture of the microtubule cytoskeleton. It is likely that work in this area has only revealed the ‘tip of the iceberg’. Better knowledge of how MAPs and motors affect the structural state of the microtubule, and the use of appropriate microtubule substrates for *in vitro* work, is necessary to understand the full impact of implications we have discussed here.
